# Supplementary material for: Analyzing efficacy, stability, and safety of AAV-mediated optogenetic hearing restoration in mice
Source: Life Sci Alliance. 2022 May 5;5(8):e202101338. doi: 10.26508/lsa.202101338 (PMC9258265; doi:10.26508/lsa.202101338)
Supplement: Supplementary file 1 [file LSA-2021-01338_TableS1.docx]

| **Age (months)** | **Injected cochlea** | | | | **Non-injected cochlea** | | | |
| --- | --- | --- | --- | --- | --- | --- | --- | --- |
|  | **Average** | **SD** | **N_animal_** | **n_slices_** | **Average** | **SD** | **N_animal_** | **n_slices_** |
| **1** | 58.9 | 26.8 | 4 | 12 | 3.48 | 5.26 | 4 | 12 |
| **3** | 32.5 | 36.2 | 3 | 9 | 2.01 | 3.72 | 3 | 9 |
| **6** | 46.6 | 31.3 | 3 | 9 | 1.43 | 1.90 | 3 | 8 |
| **12** | 42.4 | 31.1 | 6 | 18 | 3.30 | 5.54 | 6 | 18 |
| **24** | 34.1 | 31.3 | 3 | 9 | 8.05 | 14.2 | 3 | 9 |

Table S1: Average share of transduced SGN in percent across cryosections

The share of SGNs expressing f-Chrimson-eYFP expressed as percentage of all parvalbumin-positive SGNs for the injected and non-injected cochlear of the 5 age-groups investigated here. Same data of SGN cell counts as in Figure 1. In general, 3 slices per cochlea were counted (one exception due to technical reasons).
