# Supplementary material for: Analyzing efficacy, stability, and safety of AAV-mediated optogenetic hearing restoration in mice
Source: Life Sci Alliance. 2022 May 5;5(8):e202101338. doi: 10.26508/lsa.202101338 (PMC9258265; doi:10.26508/lsa.202101338)
Supplement: Supplementary file 2 [file LSA-2021-01338_TableS2.docx]

| Score | Spiral ganglion (% tissue affected) |
| --- | --- |
| 0 | None |
| 1 | <25 |
| 2 | 26-50 |
| 3 | 51-75 |
| 4 | 76-100 |

Table S2. Semiquantitative, ordinal scores based on the distribution of the spiral ganglion findings (according to Gibson-Corley et al., 2013)
