# Supplementary material for: Analyzing efficacy, stability, and safety of AAV-mediated optogenetic hearing restoration in mice
Source: Life Sci Alliance. 2022 May 5;5(8):e202101338. doi: 10.26508/lsa.202101338 (PMC9258265; doi:10.26508/lsa.202101338)
Supplement: Supplementary file 3 [file LSA-2021-01338_TableS3.docx]

**Mouse IDs per group**

|  | **1mo-v** | **3mo-v** | **3mo-pbs** | **6mo-v** | **6mo-n** | **12mo-v** | **12mo-n** | **24mo-v** |
| --- | --- | --- | --- | --- | --- | --- | --- | --- |
| #1 | 02330-16 | 1652079 | 654877 | 654918 | 652865 | 652278 | 652987 | 647605 |
| #2 | 02331-17 | 1652080 | 654878 | 654919 | 652866 | 652279 | 652991 | 647606 |
| #3 | 02332-18 | 1652081 |  | 654920 |  | 652280 |  | 647607 |
| #4 | 02333-19 |  |  |  |  |  |  |  |

**Cochlea Histology**

|  | **injected** | | **not injected** | |
| --- | --- | --- | --- | --- |
| **1mo-v** | | | | |
| **#1** | **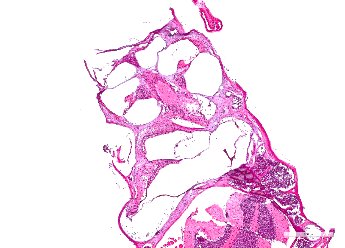** | **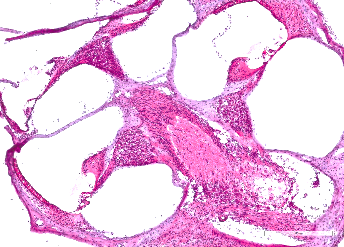** | **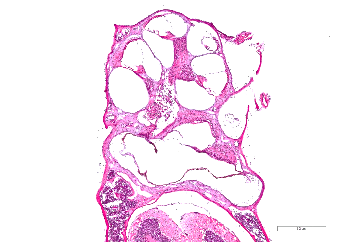** | **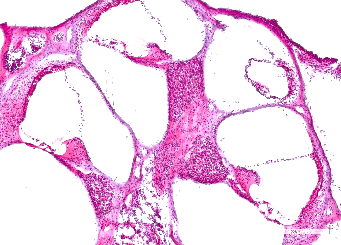** |
| **#2** | **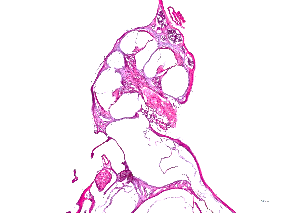** | **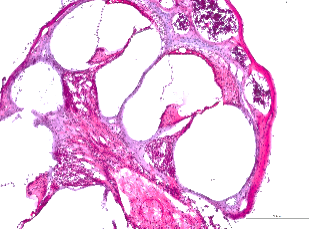** | **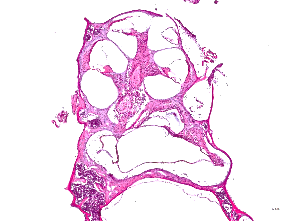** | **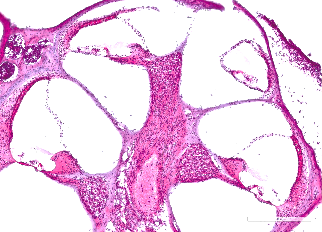** |
| **#3** | **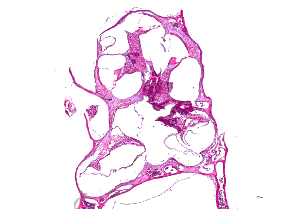** | **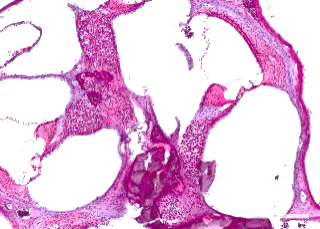** | **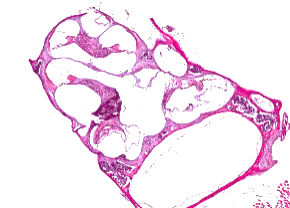** | **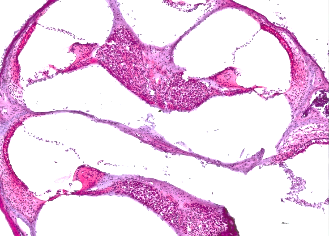** |
| **#4** | **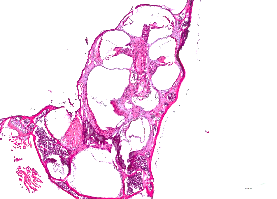** | **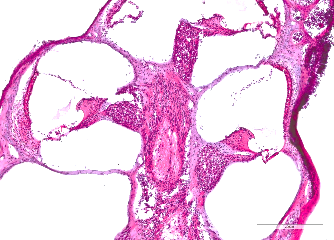** | **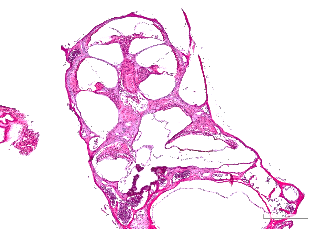** | **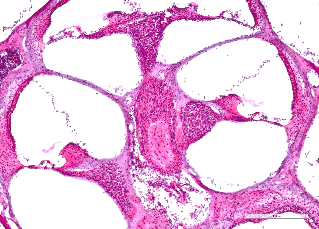** |
|  | | | | |
| **3mo-v** | | | | |
| **#1** | **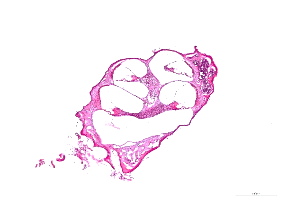** | **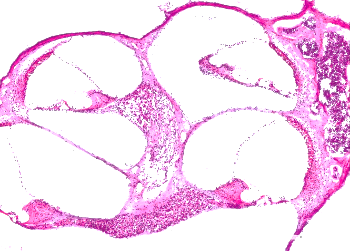** | **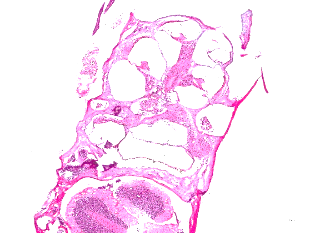** | **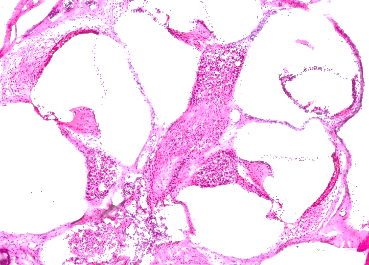** |
| **#2** | **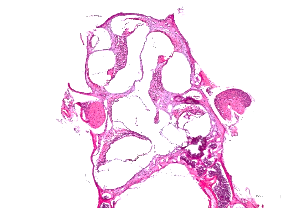** | **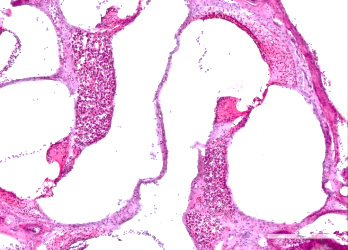** | **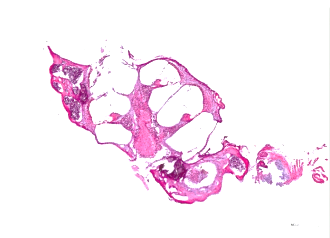** | **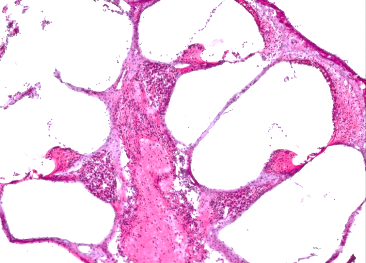** |
| **#3** | **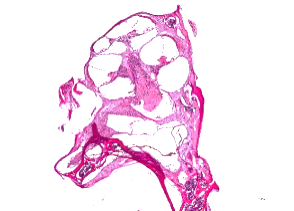** | **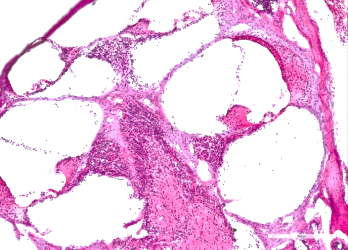** | **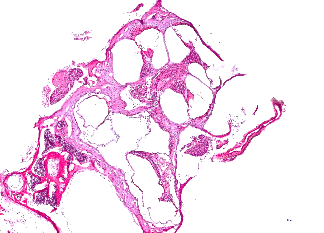** | **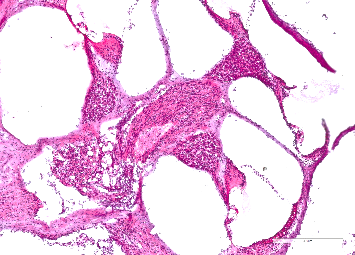** |
|  | | | | |
| **3mo-PBS** | | | | |
| **#1** | **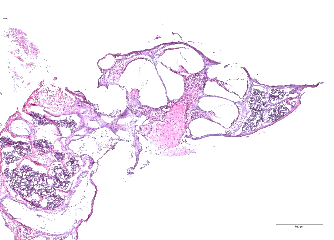** | **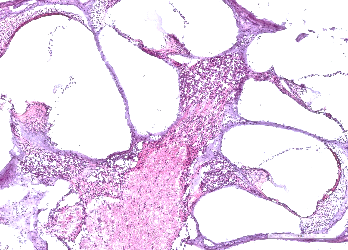** | **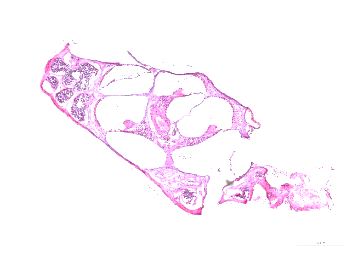** | **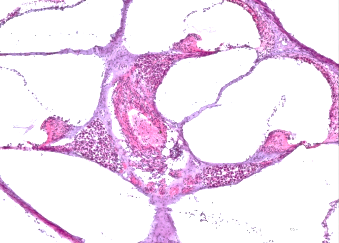** |
| **#2** | **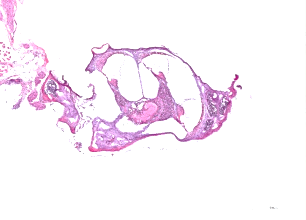** | **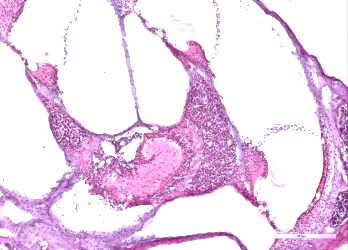** | **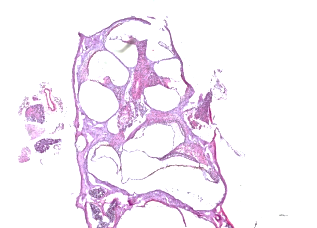** | **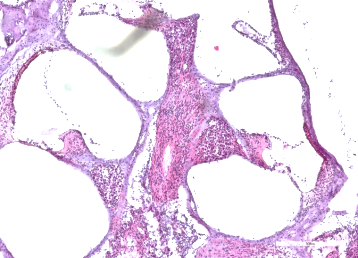** |
|  | | | | |
| **6mo-v** | | | | |
| **#1** | **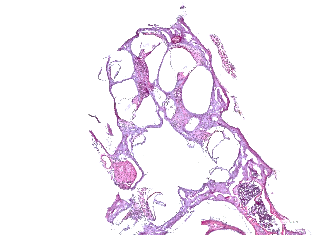** | **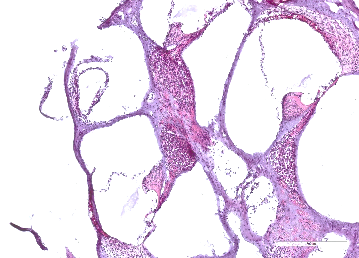** | **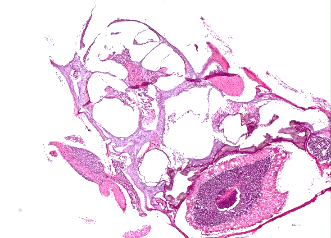** | **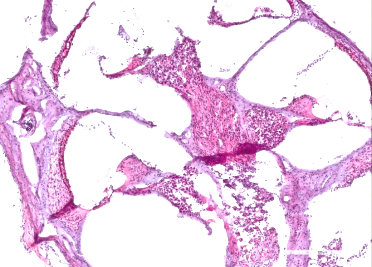** |
| **#2** | **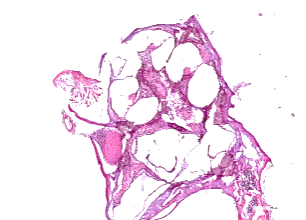** | **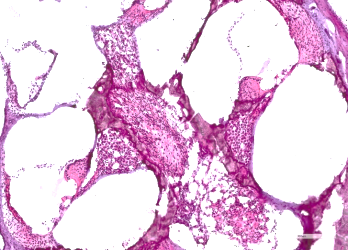** | **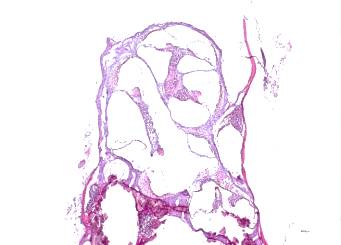** | **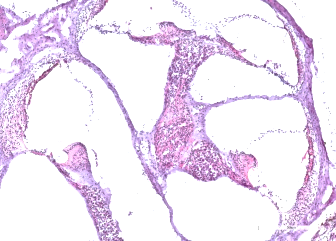** |
| **#3** | **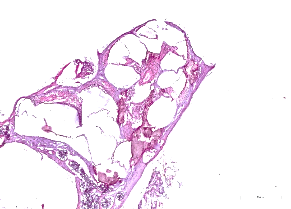** | **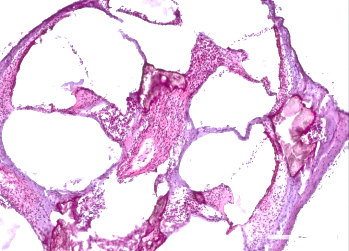** | **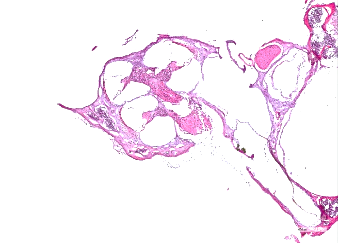** | **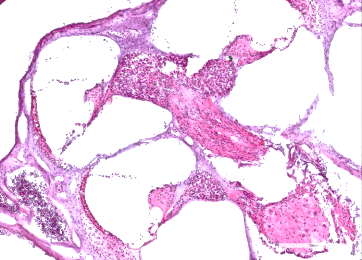** |
|  | | | | |
| **6mo-n** | | | | |
| **#1** | **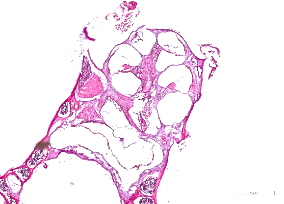** | **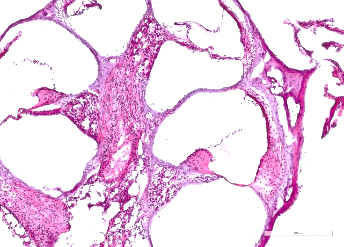** | **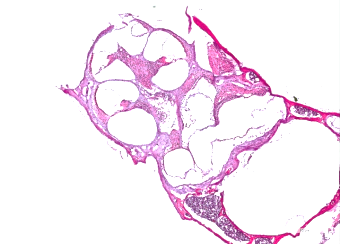** | **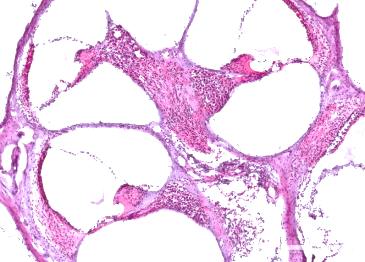** |
| **#2** | **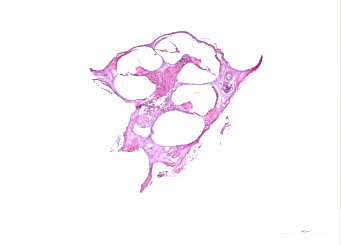** | **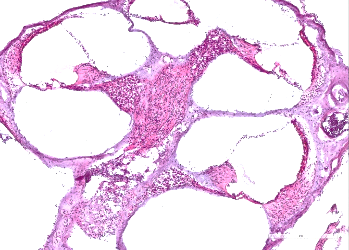** | **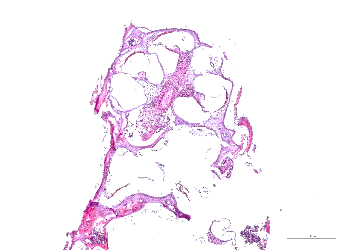** | **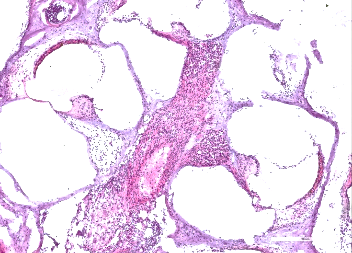** |
|  | | | | |
| **12mo-v** | | | | |
| **#1** | **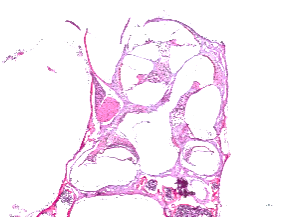** | **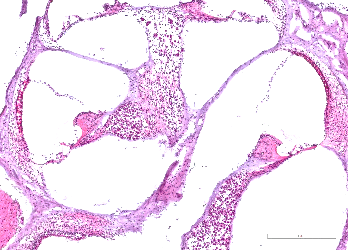** | **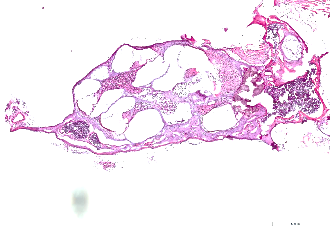** | **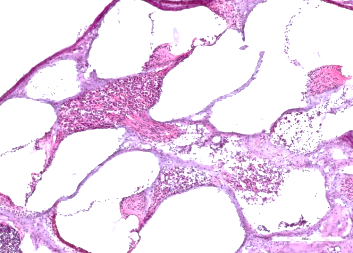** |
| **#2** | **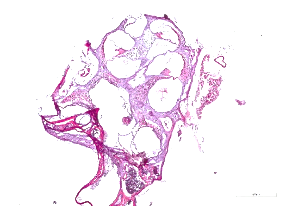** | **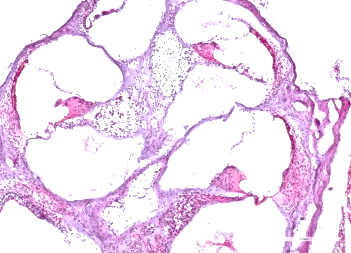** | **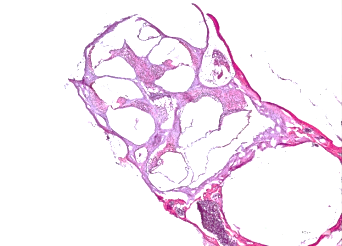** | **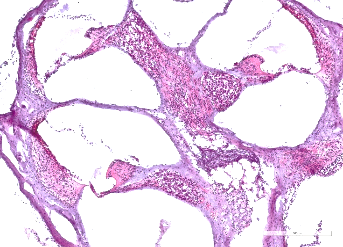** |
| **#3** | **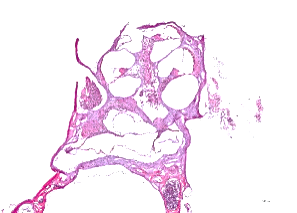** | **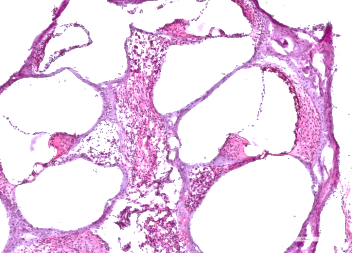** | **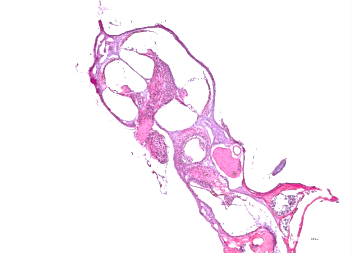** | **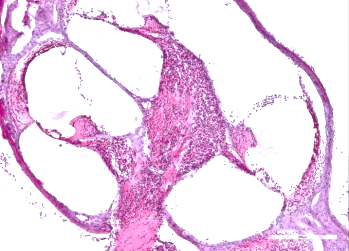** |
|  | | | | |
| **12mo-n** | | | | |
| **#1** | **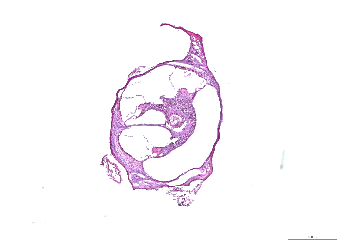** | **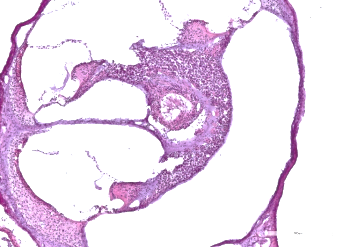** | **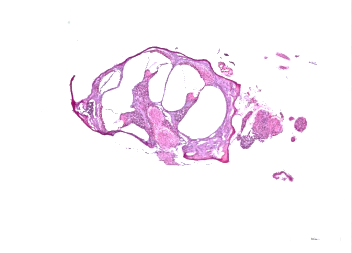** | **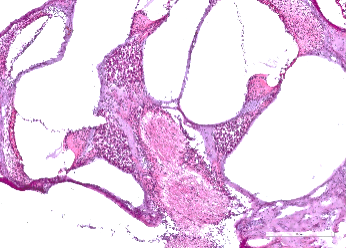** |
| **#2** | **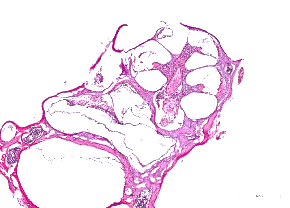** | **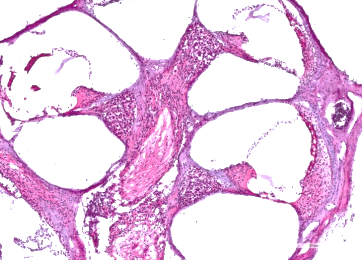** | **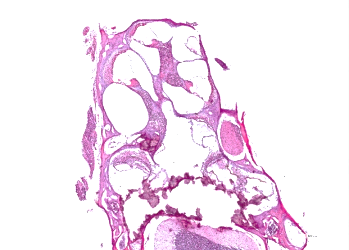** | **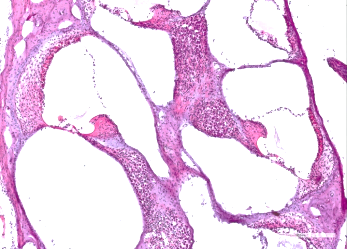** |
|  | | | | |
| **24mo-v** | | | | |
| **#1** | **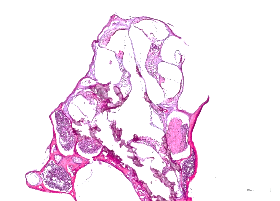** | **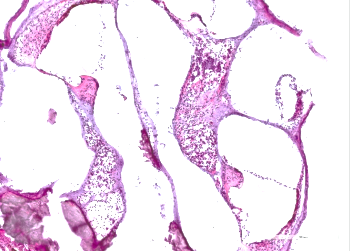** | **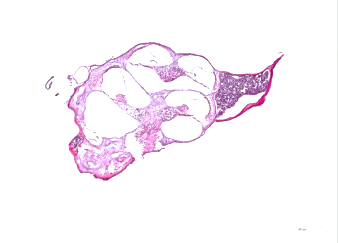** | **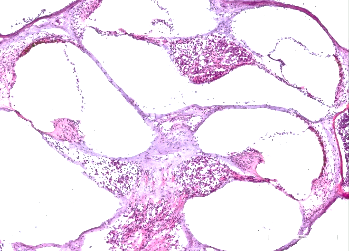** |
| **#2** | **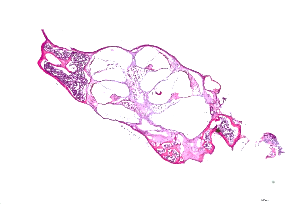** | **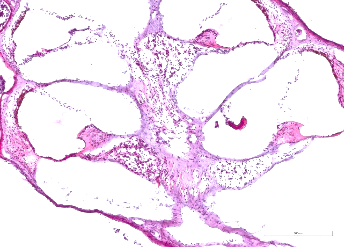** | **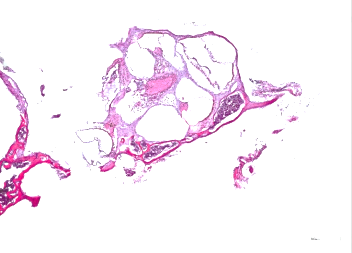** | **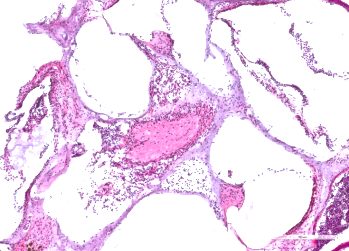** |
| **#3** | **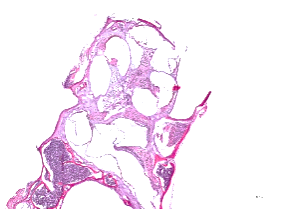** | **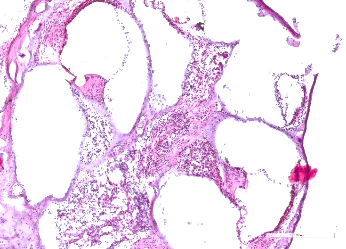** | **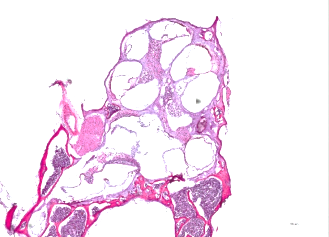** | **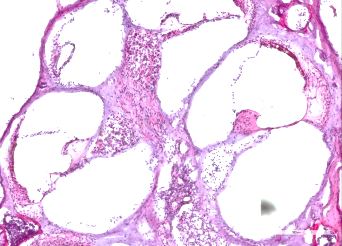** |

Table S3. Micrographs of HE stained cochleae (injected vs. non-injected side) from all mice analyzed
